# Cloning and Functional Analysis of Phosphoethanolamine Methyltransferase Promoter from Maize (*Zea mays* L.)

**DOI:** 10.3390/ijms19010191

**Published:** 2018-01-08

**Authors:** Gai-Li Niu, Wei Gou, Xiang-Long Han, Cheng Qin, Li-Xin Zhang, Abd El-Fatah Abomohra, Muhammad Ashraf

**Affiliations:** 1 State Key Laboratory of Soil Erosion and Dryland Farming on the Loess Plateau, College of Life Sciences, Northwest A&F University, Yangling 712100, China; gailiniu@nwafu.edu.cn (G.-L.N.); maokailun@nwafu.edu.cn (W.G.); hanxianglong@nwafu.edu.cn (X.-L.H.); Qincheng@nwafu.edu.cn (C.Q.); 2 New Energy Department, School of Energy and Power Engineering, Jiangsu University, Zhenjiang 212013, China; abomohra@ujs.edu.cn; 3 Botany Department, Faculty of Science, Tanta University, 31527 Tanta, Egypt; 4 Pakistan Science Foundation, Islamabad 44000, Pakistan; ashrafbot@yahoo.com

**Keywords:** maize, phosphoethanolamine *N*-methyltransferase gene (*peamt*), promoter, functional analysis

## Abstract

Betaine, a non-toxic osmoprotectant, is believed to accumulate considerably in plants under stress conditions to maintain the osmotic pressure and promote a variety of processes involved in growth and development. Phosphoethanolamine *N*-methyltransferase (PEAMT), a key enzyme for betaine synthesis, is reported to be regulated by its upstream promoter. In the present investigation, by using the transgenic approach, a 1048 bp long promoter region of *ZmPEAMT* gene from *Zea mays* was cloned and functionally characterized in tobacco. Computational analysis affirmed the existence of abiotic stress responsive *cis*-elements like ABRE, MYC, HST, LST etc., as well as pathogen, wound and phytohormone responsive motifs. For transformation in tobacco, four 5′-deletion constructs of 826 bp (P2), 642 bp (P3), 428 bp (P4) and 245 bp (P5) were constructed from the 1048 bp (P1) promoter fragment. The transgenic plants generated through a single event exhibited a promising expression of GUS reporter protein in the leaf tissues of treated with salt, drought, oxidative and cold stress as well as control plants. The GUS expression level progressively reduced from P1 to P5 in the leaf tissues, whereas a maximal expression was observed with the P3 construct in the leaves of control plants. The expression of GUS was noted to be higher in the leaves of osmotically- or salt-treated transgenic plants than that in the untreated (control) plants. An effective expression of GUS in the transgenic plants manifests that this promoter can be employed for both stress-inducible and constitutive expression of gene(s). Due to this characteristic, this potential promoter can be effectively used for genetic engineering of several crops.

## 1. Introduction

In a wide variety of plant cells, choline is a vital osmoprotectant that plays an important role in plant stress resistance by maintaining the optimal cellular activities [1]. Phosphoethanolamine *N*-methyltransferase (PEAMT) is a rate-limiting key enzyme for choline synthesis, and its main function is to catalyze the methylation of phosphoethanolamine producing phosphocholine. The latter can produce choline and phosphatidic acid under the hydrolysis of phosphocholine phosphatase, which mainly occurs in plants belonging to family Chenopodiaceae [2]. BeGora et al. [3] and Hirashima et al. [4] reported that the PEAMT three-dimensional structure is two *S*-adenosyl-l-methionine-dependent methyltransferase domains, the N-terminal (MT1) and the C-terminal (MT2) domains. Each domain contains four motifs, which could catalyze the choline synthesis. For phosphocholine (P-Cho) production, the first methylation step of phosphoethanolamine (P-Etn) to phosphomonomethylethanolamine (P-MMEtn) is catalyzed by the MT1 domain, while, the second domain (MT2) catalyzes the second methylation reaction of P-MMEtn to P-Cho [4].

It is widely reported that plant stress resistance is highly related to the PEAMT enzyme activity. The *PEAMT* gene was isolated for the first time from *Arabidopsis* [5]. Later, it was isolated from *Spinacia oleracea* [6], wheat [7], *Atriplex nummularia* L. [8], *Beta vulgaris* L. [9], and maize [10]. In a study, it was noted that the treatment of maize with 150 mmol L^−1^ NaCl caused a slight up-regulation of the expression of *ZmPEAME1* in roots, stems and leaves after 12 and 24 h [10]. However, Jost et al. [11] found that the PEAMT activity of wheat leaves and roots increased significantly under temperature stress. Their results showed that the PEAMT activity of leaves and roots increased 4 and 2 times, respectively, over the control after 6 h of temperature stress. In addition, co-cultivation of the soil bacterium *Bacillus subtilis* GB03 with *Arabidopsis* significantly enhanced the PEAMT activity, which led to an increase in the intrinsic levels of choline and betaine [12]. All the aforementioned reports confirm the effective role of enhanced PEAMF activity in stress tolerance.

Gene promoter plays a key role in gene regulation, expression and gene transcription as a significant *cis*-acting element. Li et al. [2] have obtained the promoter (−879 to +1 bp) of SlPEAMT in *S. liaotungensis* by TAIL-PCR, whose *cis*-regulatory elements included TATA-box, MYBCORE, MBS, LTR, W-box, MYC recognition site, GT-1 element, WRKY710S and LTRECOREATCOR15. Besides, some of the elements were found to be related to stress conditions. Beyond that, the *cis*-regulatory elements in the NBS-LRR resistance genes have four elements consisting of WBOX [13], DRE [14], CBF [15] and GCC box that highly related to stress [16]. Wu et al. [10] deemed that the *ZmPEAMT1* promoter contains the putative stress-responsive *cis*-acting elements, whose function have not been confirmed yet.

Studies on endogenous *PEAMT* promoters in maize need further research. In the present investigation, putative promoters from maize, which may act as potential stress-inducible promoters, were identified, cloned and characterized. They could be used in the future to propel expression of potential transgenes in crops for achieving enhanced stress tolerance. Thus, in the current investigation, for the identification of *cis*-regulatory motifs, the maize PEAMT gene putative promoter region, 1048 bp upstream from ATG, was cloned and analyzed in silico. The T1 generation transgenic tobacco plants possessing a single copy of the transgene were assessed for GUS expression under control and stressful regimes including salt, drought, cold and oxidative stress for determining the optimal length of the *ZmPEAMT* promoter for achieving effective gene expression.

## 2. Results

### 2.1. Cloning of ZmPEAMT-P and In Silico Analysis

**Figure 1:**
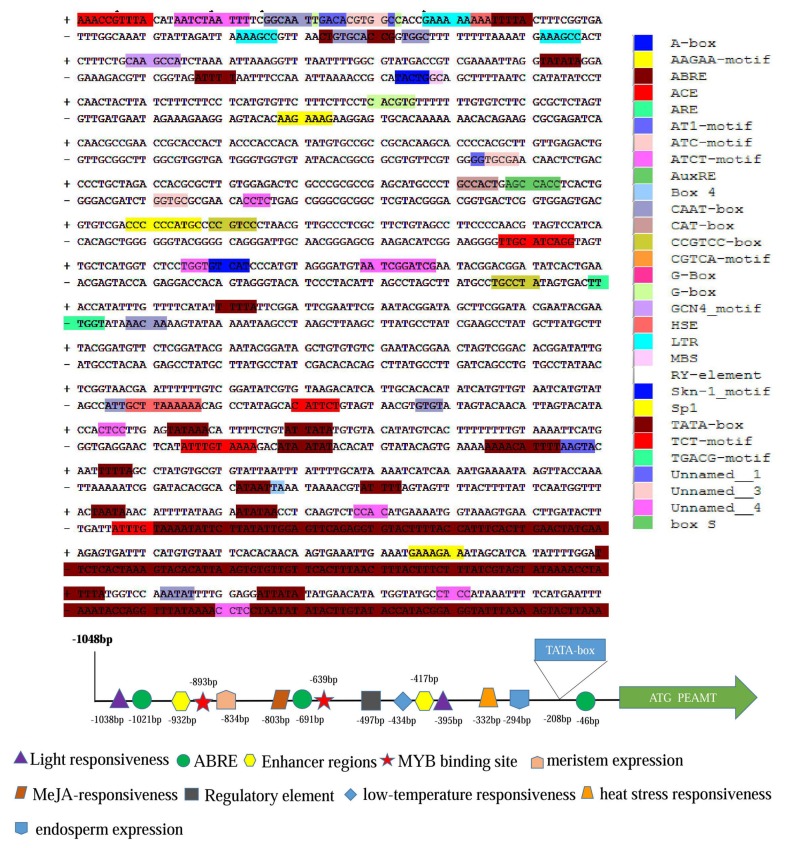
Nucleotide sequence and distribution map of *cis*-acting element of *ZmPEAMT* gene promoter in maize. Different colors and shapes represented different *cis*-acting elements of *ZmPEAMT* gene promoter.

The region of *ZmPEAMT* promoter (1048 bp) was cloned and sequenced (Figure 1) which mainly covered 10 different classes of regulatory motifs. The predicted core promoter elements included TATA-box starting from 208th bp upstream to the ATG codon and CAAT-box, which may play a key role in transcription initiation for *ZmPEAMT*. In addition, Figure 1 shows that some of *cis*-acting elements involved abiotic stress tolerance factors such as HSE, LTR, MBS, phytohormone including ABRE, AuxRE, CGTCA-motif and TGACG-motif and light regulation consisting of ACE, AT1-motif, Box4, G-Box, Sp1 and TCT-motif also existed in the sequence. Based on the analysis using the online programs PLACE and PlantCARE, the transcription initiation mechanism of *ZmPEAMT* was found to be diverse. In a nutshell, the promoter region containing core promoter elements and other *cis*-acting elements responded to abiotic stresses, light, phytohormones etc. (Table 1).

### 2.2. Promoter Deletion Constructs and Histochemical GUS Assay

**Figure 2:**
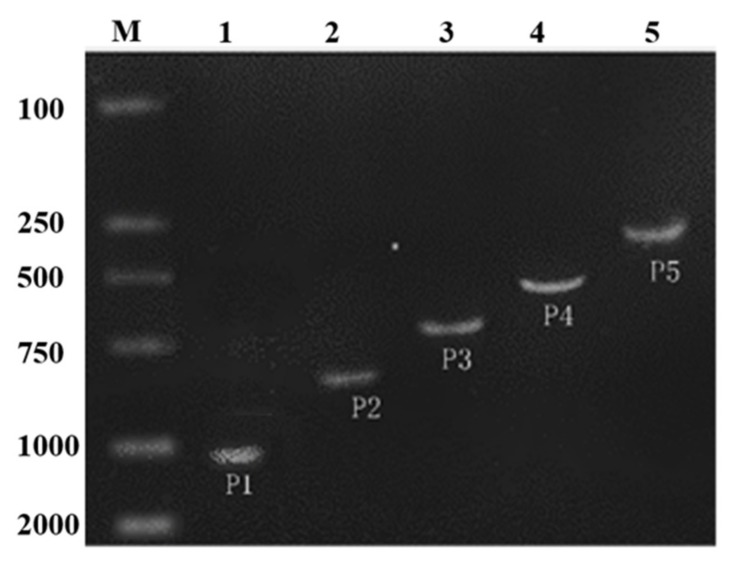
The different length of 5′-deletion of the PEAMT gene promoter in maize. The M, represented the DL2000 and 1, 2, 3, 4, 5 represented the PEAMT gene promoter P1, P2, P3, P4, P5. The size of the different promoter is 1048, 826, 642, 428 and 245 bp, respectively.

**Figure 3:**
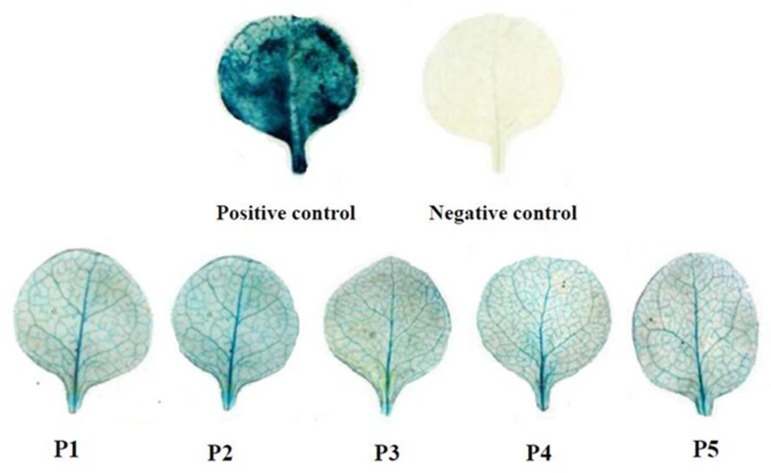
Histochemical analysis of GUS in transformed tobacco leaves with different length fragment of the PEAMT gene promoter. The positive control was transformed tobacco with pCAMBIA1301 vector. The negative control was wild type tobacco. P1 was transformed tobacco with 1048 bp promoter. P2 was transformed tobacco with 826 bp promoter. P3 was transformed tobacco with 642 bp promoter. P4 was transformed tobacco with 428 bp promoter. P5 was transformed tobacco with 245 bp promoter, respectively.

As shown in the electropherogram, the bands of P1, P2, P3, P4 and P5 were clearly observed at the corresponding position indicating the different length of 5′-deletion of the *ZmPEAMT* gene promoter in maize were cloned successfully, which was validated by gene sequencing (Figure 2). The expression vector with different length of 5′-deletion of the *ZmPEAMT* gene promoter fused with *GUS* gene was constructed and then was transformed into tobacco. The *PEAMT* gene promoter induced the *GUS* gene expression efficiently. The GUS reporter gene studied showed that the expression level of the GUS progressively decreased from P1 to P5 in the leaf tissues. As a negative control, the color of vein was white in the wild type (Wt), untransformed tobacco plants with no *GUS* gene. In contrast, the leaves of the transgenic tobacco with pCAMBIA1301 vector (positive control) appeared conspicuously blue. This phenomenon indicates the increased expression of the GUS protein in the transgenic tobacco with the pCAMBIA1301 vector. It can be observed that the vein of transgenic tobacco with *ZmPEAMT* promoters (P1, P2, P3, P4, P5) exhibited blue color of varying degree, and the most obvious was the leaves of the transgenic tobacco with vector P1, which confirms the highest expression activity (Figure 3).

### 2.3. Quantitative MUG Assays of PEAMT Gene Promoter under Abiotic Stress Conditions

**Figure 4:**
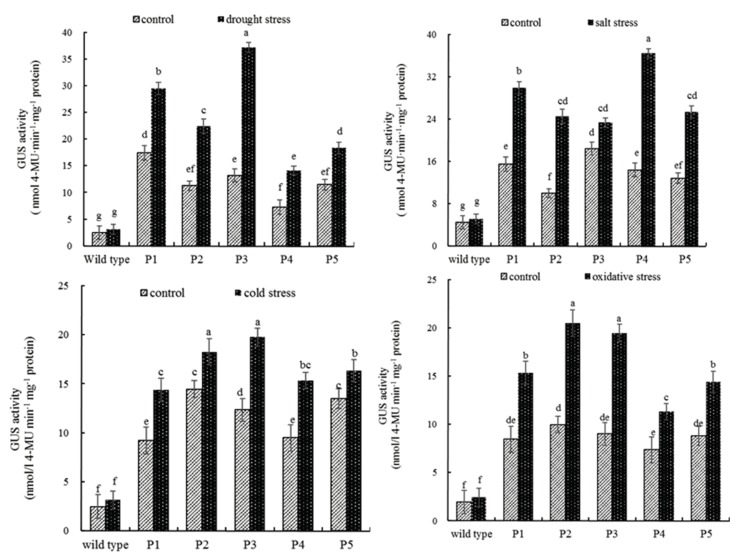
Analysis of GUS activity for PEAMT deletions in transformed tobacco leaves under different stress conditions. The drought stress condition was simulated by 15% PEG-6000 (*m*/*v*) solution. The salt stress condition was simulated by 100 mmol L^−1^ NaCl solution. The cold stress condition was simulated by 4 °C. The oxidative stress was simulated by 100 mmol L^−1^ H_2_O_2_ solution. Different letters represented significant difference among treatments at *p* < 0.05. The t-test was performed to compare differences between the inoculation treatments under drought stress conditions. Data presented as treatment means ± S.E. (*n* = 3).

In general, the transgenic plants transformed with the promoter deletion constructs exhibited marked GUS expression under stressful cues, advocating its critical function in abiotic stress tolerance by directing increased expression of the gene. Overall, the activity of the *ZmPEAMT* gene promoter varied significantly among different stressful regimes. The highest promoter activity of 39.9 nmol 4-MU min^−1^ mg^−1^ protein was recorded under saline stress, being 2.84 times higher than that of the control. Specifically, the plants transformed with P4 constructs showed a marked enhancement of the GUS expression, which represented 6.12 times higher than that of the control. Under water deficit conditions, the activities of the deletion fragment of *ZmPEAMT* gene promoter were enhanced to a varying extent compared with that of the control group. However, the activity of P3 promoter fragment was found to be the highest (37.18 nmol 4-MU min^−1^ mg^−1^ protein), which was approximately 4.76 times higher than that of the control under the saline regime. Furthermore, the plants transformed with the P3 and P2 also showed considerable GUS activity, i.e., 5.33 times and 4.85 times higher than that of the corresponding control, respectively. Under temperature stress, insignificant difference were found between the two transgenic plants. Under oxidative stress, it followed the same pattern since P2 and P3 transgenic plants showed the highest GUS activity, with insignificant difference between them (Figure 4).

## 3. Discussion

Although a variety of strategies are in vogue to improve stress tolerance in plants, the transgenic approach has recently gained a considerable interest being an efficient means of improving plant stress tolerance [17]. For generating a crop transgenic line/cultivar through genetic engineering, a pre-requisite is the availability of a potential source of gene/transgene responsible for a desired trait as well as an efficient promoter to achieve considerable degree of expression in the host plant [18]. The present study was conducted to clone a promoter of stress responsive *ZmPEAMT* gene from maize and study its functional verification in tobacco.

Numerous studies have confirmed that the *PEAMT* gene is a plant resistance-related gene, which plays a vital role in plants exposed to a variety of stress environments [1,19]. The up-regulation of the *PEAMT* gene expression under stress conditions might be attributed to the PEAMT protein belonging to a family of regulatory proteins, which are induced in the early stage of stress to help plant respond to the stimulus signal quickly [2]. The promoter, a DNA sequence located upstream of the gene, is capable of controlling the initiation of transcription and activating the RNA polymerase to bind with the template precisely [20]. In the present study, the target gene was cloned to obtain the promoter sequence at 1048 bp. Results confirmed that the transformation of tobacco mediated by *Agrobacterium tumefactions* was found to be increased under drought, salt, low temperature and oxidative stress, and the activity of GUS showed an increasing trend as compared with the control. The model of *ZmPEAMT* promoter action in transgenic tobacco assumes that abiotic stresses (salt, drought, oxidative and cold) improve the binding of stress-related transcription factors and stress response promoter elements and it increase the activity of *ZmPEAMT* promoter, which correlate with the induction of GUS expression. Tao [21] found that JcMFT1 seed-specific promoter was induced by abscisic acid because its promoter contained ABA response elements. In addition, the activity of the *SePEAMT* promoter was reported to be induced by abiotic stresses such as salt, low temperature, heat shock and ABA. PLACE and Plant CARE result showed that promoter of *ZmPEAMT* contains some stress response elements such as MBS (MYB binding site), G-box (MYC binding site), ABRE, low-temperature responsive elements etc., which may contribute to the resilience of *ZmPEAMT* gene. In another study, *Arabidopsis thaliana AtMYB2* was reported to be induced by drought stress and low temperature, and this was specifically attributed to MYB elements which improve plant resistance [22]. In addition, MYC has been reported to have a role in responding to drought [23]. G-box was shown as a binding site for MYC and it contributed to the expression of the *AtADH1* gene under drought stress [24]. ABRE is a class of elements capable of binding to the conservatively strong ABA-dependent transcription factors [25], which exist in the promoter region of many stress-resistant genes, such as those of drought, dehydration, salt and low temperature, which regulate the expression of related genes under non-biological stress [26]. Secondly, an ABRE element was reported to respond effectively to salt stress [27]. The WRKY transcription factor also had been identified as a low temperature-induced transcription factor, which is the ICE1 binding site. In addition, the expression of eight WRKY transcription factors in *Arabidopsis thaliana* was reported to be associated with the expression of cold stress-related genes under low-temperature environments [28].

Under drought stress, the activity of the *ZmPEAMT* gene promoter was improved compared with that in the control group, and the difference was markedly significant. Among them, the P3 promoter activity was changed significantly after stress treatment compared with the other fragments. According to the sequence characteristics of the *ZmPEAMT* gene promoter, four MYC binding sites (G-box) were distributed in the P3 fragment (Table 2), which may cause specific binding of some of the four acting elements to certain proteins, or the presence of drought-related positive regulators or enhancers in the region to improve the transcription level and expression activity of P3 fragment [2]. The P4 promoter had high activity under salt stress because of the existence of abscisic acid response element ABRE, etc. [29], which also play an important role in salt stress [30]. These salt stress responsive elements in this region, and the overall activity of the promoter was found to be improved under the combined action of these elements. However, the activity of the P3 promoter under salt stress was relatively low, which was presumably due to the presence of negative regulatory factors in the region to inhibit the expression of P3 fragments under salt stress. However, results of this study showed that the activity of P2 and P3 promoter fragments was relatively higher under low temperature stress. When the receptor was under low temperature stress, the gene regulated the transcription of CBF/DREB1 complex and induced the expression of cold stress related genes [31]. According to the results of the *PEAMT* gene promoter sequence, most of the transcription factors of MYB and G-box were concentrated in the first half of the sequence, suggesting that the higher activity of P2 and P3 promoter fragments under low temperature stress may be due to the interaction of these *cis*-elements. Under oxidative stress, the activity of P2 promoter fragment was relatively high in the control and stress groups, and followed by P3 in terms of its activity. The sequence analysis of the *PEAMT* gene promoter was shown that the region did not have the ability to respond to hydrogen peroxide stress, but it was still possible to detect that the promoter with an expression activity, possibly because the region had a positive regulatory factor to increase the initiation of this activity under oxidative stress. However, the specific mechanism of action still needs further verification. In general, the expression activity of P5 promoter in four different abiotic stresses was relatively low, and the possible reason could be that the promoter sequence was relatively short with lack of correlation. However, P5 promoter still had activity under most of the stress conditions, suggesting that there may have been some specific structure in the region, such as enhancers that affect the binding of RNA polymerase to the promoter, or that there were some positive regulatory elements. Mao et al. [32] found a *Py-rich* stretch element was found near the promoter close to −50 bp in *PEAMT* gene, which was reported to enhance the activity of the promoter, suggesting that the presence of this element leads to P5 promoter fragments expression under multiple stresses. P3 and P4 promoter fragments were able to regulate the efficient expression of the GUS reporter gene under most of the abiotic stress conditions, indicating that there is a *cis*-acting element that specifically regulates gene expression in this region, whereas the *ZmPEAMT* gene promoter acts as an inducible promoter, which needs to be further analyzed and validated.

Rd29A promoter, containing two stresses respond elements DRE (dehydration-responsive element), can be induced by drought, salt and low temperature, which was considered as an ideal stress-inducible promoter in the biological engineering [1]. Then Zhao et al. [33] found Rd29A promoter could drive the expression of *Arabidopsis thaliana DREB1A/CBF3* (C-repeat-binding factor) in *Festuca elata Keng ex E. Alexeev*. Under dry condition, all non-transgenic plants wilted after 30 d, but the transgenic plants wilted small part. Similarly, *ZmPEAMT* gene promoter, as a stress-inducible promoter, may be used to biological engineering for solving the problem caused by abiotic stress in different plants that might provide staple food crops.

## 4. Materials and Methods

### 4.1. Plant Materials and Growth Conditions

Seeds of maize (*Zea mays* L.) were collected from an inbred line, B73, then were sown in garden soil and cultivated in artificial climate box under 25/18 °C and 16/8 h (day/night) conditions. The leaves were collected at three leaf stages for genomic DNA extraction. Seeds of tobacco (*Nicotiana tabacum*) were sown on the nursery substrate (substrate:vermiculite = 3:1) and grown in an artificial climate chamber set at 25/18 °C air temperature and 16/8 h (day/night). The leaf tissues were used for genetic transformation at the fourth week for *ZmPEAMT* promoter analysis. The promoter of *ZmPEAMT* was transformed into tobacco induced by *Agrobacterium* mediation by means of the leaf disc method. The T1 generations were said to be the transgenic tobacco lines that contained a single insertion, whose seeds were grown in the same conditions as described earlier. After three weeks, the seedlings were transferred to a hydroponic system and two weeks later, the seedlings of the transgenic tobacco were exposed to 100 mM NaCl [34], 15% PEG-6000 (*m*/*v*) [35] and 100 mM H_2_O_2_ [36] in half strength Hoagland’s nutrient solution (24 h) for simulating salt, drought and oxidative stress, respectively. For cold stress, the seedlings were cultivated in half strength Hoagland’s solution at 4 °C [37]. The control plants were grown in Hoagland’s solution (0.5 strength) without any stress. The nutrient solution updated every four days to keep the nutrients’ concentrations almost uniform in the whole developing stage of the plants.

### 4.2. Isolation of 5′-Upstream Cis-Acting Elements and In Silico Analysis

For the extraction of genomic DNA from the leaves of maize and tobacco a modified cetyltrimethylammonium bromide (CTAB) method was employed [38]. The concentration of genomic DNA was appraised using a NanoDrop ND-1000 spectrophotometer and the quality of the genomic DNA was assessed by 1% agarose gel electrophoresis. Specific primers were designed from the *ZmPEAMT* sequence (Accession No.: 103651303) (Table 2), for the isolation of the 5′-upstream promoter region of the *ZmPEAMT* gene. The region 1048 bp upstream to the ATG from the genomic DNA of maize was amplified using standard PCR conditions which started at 95 °C (5 min) and then entered into 35 cycles of 95 °C (30 s), 50 °C (30 s), 72 °C (90 s), and then sequenced. In silico analysis, homology searches were carried out using the online programs PLACE [39] and Plant CARE [40] to identify the promoter *cis*-regulatory elements. Then they were classified into different groups based on their sequence homology [41].

### 4.3. Construction of ZmPEAMT Promoter Expression Vector and Tobacco Transformation

The construction of *ZmPEAMT* promoter vectors was constructed by taking the GUS gene with the putative *ZmPEAMT*-P instead of the CaMV35S promoter upstream according to Tiwari et al. [18]. A primer containing restriction sites HindШ and NcoI was employed to amplify the putative *ZmPEAMT*-P region upstream to ATG, then it was cloned into the pMD18-T Easy vector and confirmed by sequencing. After digesting the *ZmPEAMT*-P-T vector with HindШ and NcoI, it was sub-cloned into pCAMBIA1301, so the resultant construct was tagged as P1. Similarly, four more 5′-deletion constructs of *ZmPEAMT*-P along with the 5′-UTR were prepared using different primer sets and named P2 (826 bp), P3 (642 bp), P4 (428 bp) and P5 (245 bp), respectively. The whole promoter constructs were integrated into *Agrobacterium tumefaciens* LBA4404 and then transformed into tobacco leaf discs. Transgenic plants (T0) grown in a greenhouse and the offspring seeds were used to perform the further experiments and analysis in the next generation.

### 4.4. GUS Histochemical

The assays of GUS histochemical were implemented based on the method of Battraw and Hall [42]. The leaves that harvested from the transgenic seedlings were placed in GUS staining solution at 37 °C for 12 h. After dyeing, the samples, in turn, were put into 50%, 75% and 90% ethanol solution, 5 min each. It ended up in 100% ethanol solution until chlorophyll was completely removed. After decolorization, the leaves were re-added into ethanol solution in the order of 100%, 90%, 75% and 50% for 5 min each for rehydration, then observed under a microscope.

### 4.5. Quantitative Assay under Different Stress Conditions

For the quantitative analyses of the GUS activity, samples were triturated in 600 μL of GUS extraction buffer and centrifuged at 10,000× *g* for 15 min at 4 °C. The supernatant was used to measure the protein content, which was determined with β-Glucuronidase Reporter Gene Staining Kit (Sigma-Aldrich, St. Louis, MO, USA) using the specific steps of the kit instructions. The protein extract (50 μL) was homogenized in 450 μL of MUG buffer (the final concentration of 4-MUG was 2 mmol L^−1^) and water bath heating for 30 min at 37 °C. An aliquot of 950 μL stop buffer containing 0.2 mol L^−1^ NaCO_3_ was added to 50 μL of the extract. Then 950 μL of the stop buffer were added to 50 μL of the reaction mixture at 10, 20, 30, 40 min. The fluorescence level of 4-methylumbelliferone (4-MU), the breakdown product, was determined using a fluorescence spectrophotometer (Berthold Technologies, Germany) at excitation/emission of 365/455 nm specifically for 4-MU. The 4-MU concentration was calculated from a standard curve. The GUS activity was expressed as nmol of 4-MU min^−1^ mg^−1^ protein.

### 4.6. Statistical Analysis

All experiments were conducted in three experimental replicates. The data from the quantitative GUS assay were subjected to analysis of variance and the mean values were compared using the LSD test at 5% probability level.

## 5. Conclusions

Taken together, several abiotic stress responsive *cis*-acting elements, hormone response *cis*-acting elements, light-induced signal transduction elements, and specific activation elements involved in pollen development were detected in the *ZmPEAMT* promoter from maize. Furthermore, the presence of ABRE and MYB transcription factor binding sites on the promoter, indicates that the expression of the *PEAMT* might have been regulated by ABA-mediated signal transduction under stressful cues. An ample level of the GUS reporter protein was found in control as well as stress treated samples. Thus, the *ZmPEAMT* promoter could be effectively used to engineer crops with enhanced stress tolerance.

## Figures and Tables

**Table 1:** Details of *cis*-acting elements of the *PEAMT* gene promoter in maize.

Order	Name	Sequence	Number	Function
1	A-box	CCGTCC	3	*cis*-acting regulatory element
2	AAGAA-motif	GAAAGAA	2	
3	ABRE	GGACACGTGGC	6	*cis*-acting element involved in the abscisic acid responsiveness
		ACGTGGC		
		ACACGTGGC		
		CACGTG		
		GCAACGTGTC		
		CACGTG		
4	ACE	AAAACGTTTA	4	*cis*-acting element involved in light responsiveness
		ACTACGTTGG		
5	ARE	TGGTTT	1	*cis*-acting regulatory element essential for the anaerobic induction
6	AT1-motif	AT1-motif	1	part of a light responsive module
7	ATC-motif	TGCTATCCG	3	part of a conserved DNA module involved in light responsiveness
8	ATCT-motif	AATCTAATCT	2	part of a conserved DNA module involved in light responsiveness
		AATCTGATCG		
9	AuxRE	TGTCTCAATAAG	1	part of an auxin-responsive element
10	Box 4	ATTAAT	2	part of a conserved DNA module involved in light responsiveness
11	CAAT-box	gGCAAT	16	common *cis*-acting element in promoter and enhancer regions
		CAAT		
		CAAAT		
12	CAT-box	GCCACT	1	*cis*-acting regulatory element related to meristem expression
13	CCGTCC-box	CCGTCC	3	*cis*-acting regulatory element related to meristem specific activation
14	CGTCA-motif	CGTCA	1	*cis*-acting regulatory element involved in the MeJA-responsiveness
15	G-Box	CACGTG	2	*cis*-acting regulatory element involved in light responsiveness
16	G-box	tgACACGTGGCA	4	*cis*-acting regulatory element involved in light responsiveness
		CACGTG		
		ACACGTGGC		
17	GCN4_motif	CAAGCCA	1	*cis*-regulatory element involved in endosperm expression
18	HSE	AAAAAATTTC	2	*cis*-acting element involved in heat stress responsiveness
		AGAAAATTCG		
19	LTR	CCGAAA	5	*cis*-acting element involved in low-temperature responsiveness
20	MBS	CGGTCA	1	MYB Binding Site
21	RY-element	CATGCATG	1	*cis*-acting regulatory element involved in seed-specific regulation
22	Skn-1_motif	GTCAT	3	*cis*-acting regulatory element required for endosperm expression
23	Sp1	CC(G/A)CCC	1	light responsive element
24	TATA-box	TTTTA	35	core promoter element around −30 of transcription start
		TATA		
		TATAAA		
		ATTATA		
		TAATA		
		TACAAAA		
		TATAAAAT		
		TATAAAA		
25	TCT-motif	TCTTAC	1	part of a light responsive element
26	TGACG-motif	TGACG	1	*cis*-acting regulatory element involved in the MeJA-responsiveness
27	Unnamed_1	GCCACGTGGC	4	
		CGTGG		
28	Unnamed_3	CGTGG	3	
29	Unnamed_4	CTCC	6	
30	box S	AGCCACC	1	

**Table 2:** A list of primers used to amplify different deletions of the PEAMT gene promoter.

Primer	Sequence (5′–3′)
F1	AGCTCATCCATGCCATGTGTAATCC
F2	TAGCACCGCCTACATACCTC
F3	CGAACTGAGATACCTACAGC G
F4	TCTTTCCTGCGTTATCCC
F5	AATACGCAAACCGCCTCT
R1	TCCACACAACATACGAGCC

## Abbreviations

*PEAMT*	Phosphoethanolamine *N*-Methyltransferase
GUS	Escherichia Coli β-Glucuronidase gene
P-Etn	Phosphoethanolamine
P-Cho	Phosphocholine
P-MMEtn	Phosphomonomethylethanolamine
Wt	Wild Type
*AtADH1*	Alcohol Dehydrogenase
CTAB	Cetyltrimethylammonium Bromide

## References

[1] Scholz A., Stahl J., De B.V., Müller V., Averhoff B. (2016). Osmotic stress response in acinetobacter baylyi: Identification of a glycine-betaine biosynthesis pathway and regulation of osmoadaptive choline uptake and glycine-betaine synthesis through a choline-responsive BetI repressor. Env. Microbiol. Rep..

[2] Li Q.L., Xie J.H., Ma X.Q., Li D. (2016). Molecular cloning of Phosphoethanolamine *N*-methyltransferase (PEAMT) gene and its promoter from the halophyte Suaeda liaotungensis and their response to salt stress. Acta Physiol. Plant.

[3] BeGora M.D., Macleod M.J.R., McCarry B.E., Summers P.S., Weretilnyk E.A. (2010). Identification of phosphomethylethanolamine *N*-methyltransferase from Arabidopsis and its role in choline and phospholipid metabolism. J. Biol. Chem..

[4] Hirashima T., Toyoshima M., Moriyama T., Nakamura Y., Sato N. (2017). Characterization of phosphoethanolamine-*N*-methyltransferases in green algae. Biochem. Bioph. Res. Commun..

[5] Bolognese C.P., Mcgraw P. (2000). The isolation and characterization in yeast of a gene for Arabidopsis S-adenosylmethionine: Phospho-ethanolamine *N*-methyltransferase. Plant Physiol..

[6] Nuccio M.L., Ziemak M.J., Henry S.A., Weretilnyk E.A., Hanson A.D. (2000). cDNA cloning of phosphoethanolamine *N*-methyltransferase from Spinach by complementation in Schizosac charomyces pombe and characterization of the recombinant enzyme. J. Biol. Chem..

[7] Charron J.B.F., Breton G., Danyluk J., Muzac I., Ibrahim R.K., Sarhan F. (2002). Molecular and biochemical characterization of a cold-regulated phosphoethanolamine *N*-methyltransferase from wheat. Plant Physiol..

[8] Tabuchi T., Kawaguchi Y., Azuma T., Nanmori T., Yasuda T. (2005). Similar regulation patterns of choline monooxygenase, phosphoethanolamine *N*-methyltransferase and *S*-adenosyl-l-methionine synthetase in leaves of the halophyte *Atriplex nummularia* L.. Plant Cell Physiol..

[9] Tabuchi T., Okada T., Takashima Y., Azuma T., Nanmori T., Yasuda T. (2006). Transcriptional response of glycine betaine-related genes to salt stress and light in leaf beet. Plant Biotechnol. J..

[10] Wu S., Yu Z., Wang F., Li W., Ye C., Li J., Tang J.H., Ding J.Q., Zhao J.R., Wang B. (2007). Cloning, characterization, and transformation of the phosphoethanolamine *N*-methyltransferase gene (ZmPEAMT) in maize (*Zea mays* L.). Mol. Biotechnol..

[11] Jost R., Berkowitz O., Shaw J., Masle J. (2009). Biochemical characterization of two wheat phosphoethanolamine *N*-methyltransferase isoforms with different sensitivities to inhibition by phosphatidic acid. J. Biol. Chem..

[12] Zhang H., Murzello C., Sun Y., Kim M.S., Xie X., Jeter R.M., Zak J.C., Dowd S.C., Paré P.W. (2010). Choline and osmotic-stress tolerance induced in Arabidopsis by the soil microbe Bacillus subtilis (GB03). Mol. Plant Microbe Interact..

[13] Dong J., Chen C., Chen Z. (2003). Expression profiles of the arabidopsis wrky gene superfamily during plant defense response. Plant Mol. Biol..

[14] Sakuma Y., Maruyama K., Osakabe Y., Qin F., Seki M., Shinozaki K., Yamaguchi-Shinozakia K. (2006). Functional analysis of an Arabidopsis transcription factor, DREB2A, involved in drought-responsive gene expression. Plant Cell.

[15] Ohmetakagi M., Suzuki K., Shinshi H. (2000). Regulation of ethylene-induced transcription of defense genes. Plant Cell Physiol..

[16] Sharma R., Rawat V., Suresh C.G. (2017). Genome-wide identification and tissue-specific expression analysis of nucleotide binding site-leucine rich repeat gene family in Cicer arietinum (kabuli chickpea). Genomics Data..

[17] BhatnagarMathur P., Vadez V., Sharma K.K. (2008). Transgenic approaches for abiotic stress tolerance in plants: Retrospect and prospects. Plant Cell Rep..

[18] Tiwari V., Patel M.K., Chaturvedi A.K., Mishra A., Jha B. (2016). Functional Characterization of the Tau class glutathione-s-transferases gene (SbGSTU) promoter of Salicornia brachiata under salinity and osmotic stress. PLoS ONE.

[19] Ye C., Wu S., Yang Q., Ma C., Yang G., Wang B. (2005). Cloning, sequencing and salt induced expression of peamt and badh in oilseed rape (brassica napus). DNA Seq..

[20] Hernandez-Garcia C.M., Finer J.J. (2014). Identification and validation of promoters and cis-acting regulatory elements. Plant Sci..

[21] Tao Y.B., Luo L., He L.L., Ni J., Xu Z.F. (2014). A promoter analysis of mother of ft and rft11 (JcMFT1), a seed-preferential gene from the biofuel plant Jatropha curcas. J. Plant Res..

[22] Peng X., Liu H., Wang D., Shen S. (2016). Genome-wide identification of the *Jatropha curcas* MYB family and functional analysis of the abiotic stress responsive gene JcMYB2. BMC Genom..

[23] Roy S. (2016). Function of MYB domain transcription factors in abiotic stress and epigenetic control of stress response in plant genome. Plant Signal. Behav..

[24] Bruxelles G.L.D., Peacock W.J., Dennis E.S., Dolferus R. (1996). Abscisic Acid Induces the Alcohol Dehydrogenase Gene in Arabidopsis. Plant Physiol..

[25] Guiltinan M.J., Marcotte W.R., Quatrano R.S. (1990). A plant leucine zipper protein that recognizes an abscisic acid response element. Science.

[26] Narusaka Y., Nakashima K., Shinwari Z.K., Sakuma Y., Furihata T., Abe H., Narusaka M., Shinozaki K., Yamaguchi-Shinozaki K. (2003). Interaction between two cis-acting elements, ABRE and DRE, in ABA-dependent expression of Arabidopsis rd29A gene in response to dehydration and high-salinity stresses. Plant J..

[27] Park S.H., Bang S.W., Jeong J.S., Jung H., Redillas M.C.F.R., Kim H.I., Lee L.H., Kim Y.S., Kim J.K. (2012). Analysis of the APX, PGD1 and R1G1B constitutive gene promoters in various organs over three homozygous generations of transgenic rice plants. Planta.

[28] Banerjee A., Roychoudhury A. (2015). WRKY proteins: Signaling and regulation of expression during abiotic stress responses. Sci. World J..

[29] Fujita Y., Yoshida T., Yamaguchi-Shinozaki K. (2013). Pivotal role of the AREB/ABF-SnRK2 pathway in ABRE-mediated transcription in response to osmotic stress in plants. Physiol. Plantarum..

[30] Kakali M., Roy C.A., Bhaskar G., Sudhiranjan G., Sengupta D. (2006). An abre-binding factor, osbz8, is highly expressed in salt tolerant cultivars than in salt sensitive cultivars of indica rice. BMC Plant Biol..

[31] Chinnusamy V., Schumaker K., Zhu J.K. (2004). Molecular genetic perspectives on cross-talk and specificity in abiotic stress signalling in plants. J. Exp. Bot..

[32] Mao X., Jia D., Li A., Zhang H., Tian S., Zhang X., Jing R. (2011). Transgenic expression of TaMYB2A confers enhanced tolerance to multiple abiotic stresses in Arabidopsis. Funct. Integr. Genomic..

[33] Zhao J., Ren W., Zhi D., Wang L., Xia G. (2007). Arabidopsis dreb1a/cbf3 bestowed transgenic tall fescue increased tolerance to drought stress. Plant Cell Rep..

[34] Cao H., Wang L., Nawaz M.A., Niu M., Sun J., Xie J., Kong Q., Huang Y., Cheng F., Bie Z. (2017). Ectopic expression of pumpkin nac transcription factor cmnac1 improves multiple abiotic stress tolerance in arabidopsis. Front. Plant Sci..

[35] Yang H., Zhao L., Zhao S., Wang J., Shi H. (2017). Biochemical and transcriptomic analyses of drought stress responses of LY1306 tobacco strain. Sci. Rep..

[36] Wang W., Wang Y., Du Y., Zhao Z., Zhu X., Jiang X., Shu Z., Yin Y., Li X. (2014). Overexpression of camellia sinensis h1 histone gene confers abiotic stress tolerance in transgenic tobacco. Plant Cell Rep..

[37] Liu Y., Wang L., Zhang T., Yang X., Li D. (2017). Functional characterization of ks-type dehydrin zmdhn13 and its related conserved domains under oxidative stress. Sci. Rep..

[38] Siegel C.S., Stevenson F.O., Zimmer E.A. (2017). Evaluation and comparison of fta card and ctab dna extraction methods for non-agricultural taxa1. Appl. Plant Sci..

[39] Higo K., Iwamoto M., Higo H. (1998). Place: A database of plant cis-acting regulatory DNA elements. Nucleic Acids Res..

[40] Lescot M., Déhais P., Thijs G., Marchal K., Moreau Y., Van D., Rouze P., Rombauts S. (2002). Plantcare, a database of plant cis-acting regulatory elements and a portal to tools for in silico analysis of promoter sequences. Nucleic Acids Res..

[41] Higo K., Ugawa Y., Iwamoto M., Korenaga T. (1999). Plant cis-acting regulatory DNA elements (PLACE) database. Nucleic Acids Res..

[42] Battraw M.J., Hall T.C. (1990). Histochemical analysis of CaMV35S promoter-β-glucuronidase gene expression in transgenic rice plants. Plant Mol. Biol..

